# Using transient, effector-specific neural responses to gate decoding for brain–computer interfaces

**DOI:** 10.1088/1741-2552/adaa1f

**Published:** 2025-02-11

**Authors:** Brian M Dekleva, Jennifer L Collinger

**Affiliations:** 1 Rehab Neural Engineering Labs, University of Pittsburgh, Pittsburgh, PA, United States of America; 2 Physical Medicine and Rehabilitation, University of Pittsburgh, Pittsburgh, PA, United States of America; 3 Bioengineering, University of Pittsburgh, Pittsburgh, PA, United States of America; 4 Center for the Neural Basis of Cognition, Pittsburgh, PA, United States of America; 5 Biomedical Engineering Department, Carnegie Mellon University, Pittsburgh, PA, United States of America

**Keywords:** brain–computer interfaces, decoding, transients, grasp

## Abstract

*Objective.* Real-world implementation of brain–computer interfaces (BCIs) for continuous control of devices should ideally rely on fully asynchronous decoding approaches. That is, the decoding algorithm should continuously update its output by estimating the user’s intended actions from real-time neural activity, without the need for any temporal alignment to an external cue. This kind of open-ended temporal flexibility is necessary to achieve naturalistic and intuitive control. However, the relation between cortical activity and behavior is not stationary: neural responses that appear related to a certain aspect of behavior (e.g. grasp force) in one context will exhibit a relationship to something else in another context (e.g. reach speed). This presents a challenge for generalizable decoding, since the applicability of a decoder for a given parameter changes over time. *Approach.* We developed a method to simplify the problem of continuous decoding that uses transient, end effector-specific neural responses to identify periods of relevant effector engagement. Specifically, we use transient responses in the population response observed at the onset and offset of all hand-related actions to signal the applicability of hand-related feature decoders (e.g. digit movement or force). By using this transient-based gating approach, specific feature decoding models can be simpler (owing to local linearities) and are less sensitive to interference from cross-effector interference such as combined reaching and grasping actions. *Main results.* The transient-based decoding approach enabled high-quality online decoding of grasp force and individual finger control in multiple behavioral paradigms. The benefits of the gated approach are most evident in tasks that require both hand and arm control, for which standard continuous decoding approaches exhibit high output variability. *Significance.* The approach proposed here addresses the challenge of decoder generalization across contexts. By limiting decoding to identified periods of effector engagement, this approach can support reliable BCI control in real-world applications.

Clinical Trial ID: NCT01894802

## 1. Introduction

Most of the research related to the cortical control of movement—and by extension, brain–computer interface (BCI) control—is conducted within highly controlled laboratory settings, using a very limited set of actions. The resulting datasets, which contain only a small subset of the total repertoire of possible motor behaviors, require researchers to assume that movement-related variables (kinematics, kinetics, etc) are represented in cortical activity by a somewhat straightforward, time-invariant coding scheme. However, recent experimental paradigms that introduce even the slightest bit of complexity—multiple tasks, free behavior, etc—reveal a more complex relationship (Downey *et al* 2018, Altan *et al* 2023). For example, neural correlates of grasp force are readily decodable from motor cortex during grasp-only tasks, but disappear during periods of simultaneous arm movement (Downey *et al* 2018).

**Figure 1.**
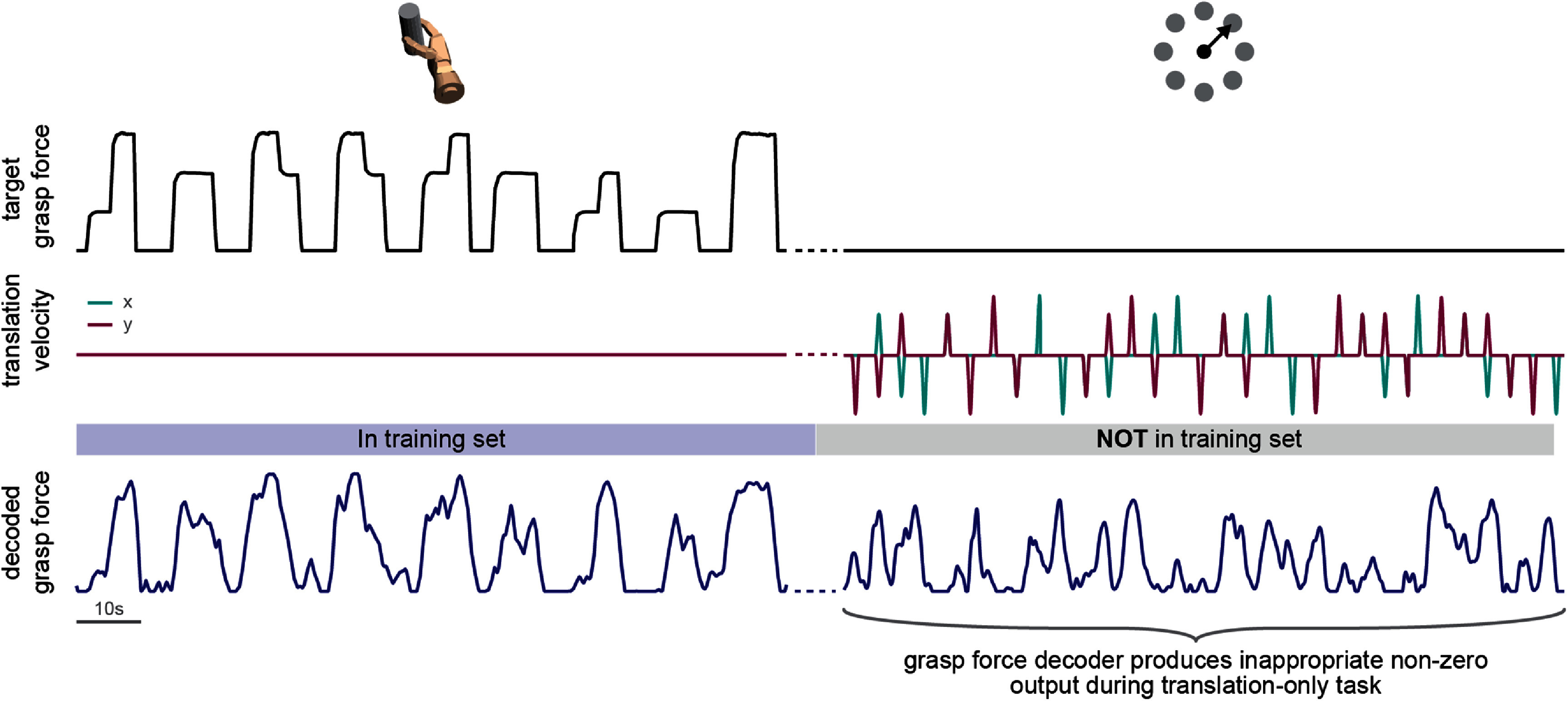
Example of poor BCI decoder generalization across behaviors. Left and right sides show traces for grasp force (top) and translation velocity (middle) after concatenating grasp-only and center-out reaching tasks performed during a single session. The bottom trace shows the decoded grasp force during both tasks using a Wiener cascade trained only on the grasp-only task data. The nonzero grasp force predictions during the reaching task demonstrate the issue of generalization, where neural activity for tasks outside of the training data induces incorrect phantom predictions.

The fact that the relationship between cortical activity and a given movement variable is nonstationary and contextual, shifting with the addition of simultaneous movements/tasks, is problematic for achieving robust BCI performance. Ideally, there would be a single correlation pattern in neural firing that accurately and consistently mapped to some degree of freedom of control. This ‘labeled line’ coding scheme would allow for consistent decoding of intent regardless of the broader behavioral context. Unfortunately, cortical control appears to be much more complex in its organization. Neural activity that seems highly correlated with a given component of control in one moment might then seem related to a completely different component the next. This problem is illustrated in figure 1, where a simple linear regression-based decoder trained to decode grasp force on a grasp-only BCI task (left) is applied minutes later (offline) to neural data acquired during a center-out BCI reaching task. Since the neural activity patterns generated during attempted reaching actions are not wholly distinct from those during hand grasp, the decoder produces strong (inappropriate) grasp force predictions. This illustrates the inherent difficulty in creating decoders that will function across different behavioral contexts, especially when extrapolating to situations not included in the training dataset.

How then should we approach BCI decoder development for generalizable use, given that we cannot count on a static, unique relationship between neural activity and a single movement parameter? One approach would be to vastly increase the complexity of the decoder model. Most BCI demonstrations have used simple linear models, but there has been a growing interest in large, neural network-based control models (Qi *et al* 2019, Zou *et al* 2021, Azabou *et al* 2023, Xiao *et al* 2023, Ye *et al* 2023). It is unclear whether these complex architectures will allow for a significant jump in generalization while maintaining a high level of performance for any given task. If they do, it will certainly come at the cost of massively larger training sets, with behavioral data that spans a much larger range of the full behavioral repertoire. With a large enough training set, the problem of generalization becomes one of interpolation, for which large network-based approaches seem well-suited. Currently, though, the simple, low-dimensional nature of laboratory tasks make the problem one of extrapolation, which is inherently more difficult.

Rather than attempt to tackle the problem of poor generalizable control using increasingly complex models and data, we took a different approach. Instead of decoding variables continuously, we broke the problem of decoding into two parts: end-effector detection and action decoding. Fundamentally, this approach was based on three key observations regarding the cortical control of movement: (1) motor cortex activity appears to be more strongly related to *changes* in motor output than to static outputs (Georgopoulos and Massey 1985, Shalit *et al* 2012), (2) for even the simplest behaviors, cortical activity is multi-dimensional and contains ‘nonspecific’, often transient components (Kobak *et al* 2016, Gallego *et al* 2018, Dekleva *et al* 2021), and (3) for short time epochs and isolated behavioral contexts, the relationship between neural activity and many movement parameters is approximately linear (Gao *et al* 2003, Sachs *et al* 2015, Naufel *et al* 2019, Glaser *et al* 2020). Based on these observations, we developed a novel method for decoding hand-related movement parameters, namely finger classification and grasp force. The core approach of the method is to detect onset and offset of a hand-related action, and only engage specific hand-related decoders (e.g. for finger and/or force) during a small temporal window at action onset. By restricting the time period of hand-related decoder engagement to brief temporal epochs, we avoid many of the common issues of continual decoding. This gated decoding approach comes at the cost of precise moment-by-moment decoding but shows promise as a useful architecture for providing robust, clinically useful control for applications such as click-and-drag computer cursor use and prosthetic hand grasp.

## 2. Methods

### 2.1. Subjects

This study contains data collected from two participants (P2 and P3) in an ongoing clinical trial of an intracortical sensorimotor BCI conducted under an FDA Investigational Device Exemption (registered at ClinicalTrials.gov NCT01894802). The study was conducted in accordance with the ethical principles outlined in the Declaration of Helsinki and approved by the Institutional Review Board at the University of Pittsburgh (Pittsburgh, Pennsylvania). P2 is a 36 year-old man with tetraplegia caused by C5 ASIA B spinal cord injury. P3 is a 31 year-old man with tetraplegia caused by incomplete C6 ASIA A spinal cord injury. Both participants retain some residual upper arm and wrist movement, but no hand function.

Both participants had two microelectrode arrays (Blackrock Microsystems, Salt Lake City, UT) implanted in the hand and arm areas of motor cortex (P2: two 88-channel arrays, P3: two 96-channel arrays). They also had two 64-channel arrays implanted in somatosensory cortex (Flesher *et al* 2016), which were not used for this study. Informed consent was obtained prior to performing any study-related procedures. Data from P2 spans from one to nine years post-implant, and data for P3 from one month to four years post-implant.

### 2.2. Data acquisition

We collected neural data both with both analog patient cables and digital NeuroPlex E headstages connected via fiber optic cable to two synced Neural Signal Processors (Blackrock Microsystems). The neural signals were filtered using a 4th order 250 Hz high-pass filter, logged as threshold crossings (−4.5 RMS) and subsequently binned at 50 Hz. These binned counts were then square root transformed and convolved offline with a causal, 400 ms decaying exponential filter to provide a smoothed estimate of firing rate.

### 2.3. Behavioral tasks

We investigated the content of the population-wide neural responses across four main types of tasks: grasp force, grasp force + carry, finger click, and finger click + drag. Each session of a given task type consisted of approximately 40 trials, where the behavior (on-screen hand grasp, button click, etc) was controlled by the computer and the participant imagined following along by covertly performing the cued action. The specific timing of the trial progression could vary across sessions, but an outline of each task type is given below.

#### 2.3.1. Grasp force

On a monitor, the participants observed a virtual hand (MuJoCo, DeepMind Technologies Limited) and cylinder. At the start of each trial, they received an audio cue signaling the desired grasp force level (e.g. ‘gentle’ or ‘firm’). After a brief delay, a tone played and the hand grasped the object, maintaining it until a subsequent tone and hand release. The participants attempted to perform grasps with their paralyzed hands in time with the displayed action. We analyzed 62 grasp force sessions for P2 (48 with two force levels and 14 with four force levels) and 46 for P3 (41 with two force levels and 5 with four force levels).

#### 2.3.2. Grasp force + carry

The participants observed a virtual hand (MuJoCo) and cylinder positioned on a tabletop to either the left or right side. At the start of each trial, the hand moved to the cylinder’s location and an audio cue signaled the desired grasp force (e.g. ‘gentle’ or ‘firm’). The hand then grasped the cylinder and, after a subsequent tone, translated it to a target location on the other side of the tabletop and released it. The participants attempted to perform grasps at the cued force levels with their paralyzed hands in time with the displayed actions. Since both participants retained some upper limb function, they covertly imagined performing the translation actions, but did not actually move their arms. We analyzed 18 grasp force + carry sessions for P2 (all with two force levels) and 10 for P3 (all with two force levels).

#### 2.3.3. Finger click

The participants observed five open circles on a screen in front of them, representing each of the five fingers. At the start of each trial, an audio cue indicated the upcoming digit (‘index’, ‘thumb’, etc), after which the corresponding circle became filled and a ‘click’ sound played. After a brief hold (variable, but usually 1–2 s) an audio cue played (‘release’) and the circle became open again. The participants attempted to perform individual finger presses in time with the displayed button ‘clicks’. We analyzed 13 finger click sessions for P2 (1 four-finger and 12 five-finger) and 7 for P3 (1 three-finger and 6 five-finger).

#### 2.3.4. Finger click + drag

On a monitor in front of them, the participants observed a cursor performing a modified eight-target center-out task. At the start of each trial, a target appeared at one of eight locations on the periphery. After a ‘go’ tone, the cursor (an open circle) moved to the target. An audio cue then indicated the upcoming click type (e.g. ‘index’) and the cursor became filled, accompanied by a ‘click’ sound. The fill color of the cursor during click depended on the click type. Once clicked, the central target appeared, and the cursor moved to it. An audio cue then signaled ‘release’, and the cursor became unfilled once more. The participants attempted to perform finger presses/releases in time with the observed cursor clicks/unclicks. As for the grasp force + carry task, they imagined upper arm translation to match the movement of the cursor. We analyzed 14 finger click + drag sessions for P2 (9 two-digit, 3 three-digit, and 2 four-digit). P3 did not perform any finger click + drag sessions.

### 2.4. Transient component identification

We identified transient components within the population activity corresponding to grasp (or finger press) onset and offset, we first performed dimensionality reduction of the firing rates from the 176 (P2) and 192 (P3) channels recording from motor cortex using factor analysis (20 factors). We then used singular value decomposition on the factor loadings to create an orthonormal latent space.

For each session, we aligned the latent responses to action onset (−1.5 s before to 2.0 s after) and offset (−2.0 s before to 1.5 s after) and averaged across trials. We then performed an initial subspace decomposition in which we identified subspaces that contained onset-unique or offset-unique variance. Within each of these subspaces, we then performed a varimax rotation on the multi-dimensional average latent response, which serves to identify projections that highlight temporally sparse responses. The specifics of this subspace decomposition and varimax approach can be found in Dekleva *et al* (2024). Once we identified dimensions containing these putative onset- and offset-specific responses, we then selected the single component that exhibited the largest modulation during onset (time window specified above) and the component with the largest modulation during offset. Due to the subspace decomposition, the two identified dimensions occupied orthogonal dimensions of the 20D latent space.

### 2.5. Condition-dependent variance

We aimed to quantify the amount of condition-dependent variance at each time point relative to action onset and offset. Here ‘condition’ refers to either the intended grasp force or the specific finger, depending on the task type. To do this, we first aligned the 20D latent activity identified from the motor cortical activity to the cued onset and offset times, just as we did when determining the transient components (see *Transient component identification*). Then, for each bin we calculated the variance across the average condition means. For example, for a five-finger pressing task, we calculated the average latent state for each of the five fingers at time *t* and then computed the total variance across these five 20D points.

### 2.6. Online decoding using gated approach

As validation of the approach, we include examples of online decoder outputs for each of the four task types. The online tasks closely mirrored those described above, (see **Behavioral tasks)**, with some slight modifications. For the online grasp force task, the participant observed a side-scrolling line trace indicating the decoded force level. A horizontal line at the start of each trial indicated the target force level. For the grasp force + carry task, the participant did not receive any moment-by-moment feedback of grasp force. Instead, the hand position was fixed at the cylinder until the grasp force reached a level within some threshold of the target force, at which point translation control (one-dimensional) was enabled. The participant was asked to transport the cylinder back and forth across the table five times and then place in on a target location and release. For the finger click task, a trial began by highlighting one of the circles (line width increase). The participant then attempted to press the associated finger (circle became filled) and release (circle returned to open). Generally, he was instructed to progress through trials as quickly as possible, except for a few trials in which we explicitly asked him to extend the duration of the click period. Finally, the finger click + drag task took the form of a gamified center-out, similar to the one described in Dekleva *et al* (2021). Briefly, the participant controlled a cursor with attached helicopter graphic to peripheral targets (smiley faces), click them, and drag them back to the central target before releasing. The color of the outer targets indicated the click type required to pick them up.

### 2.7. Offline decoding comparisons

To characterize performance of the gated decoding approach as compared to existing continuous decoding approaches, we performed an offline decoding comparison. For each case, we held out each trial, trained a decoder on the remaining trials from the same session, and then applied the decoder to the held-out trial data. We used this leave-one-out cross-validation approach for the gated approach, standard Wiener cascade (for grasp force tasks), and standard linear discriminant analysis (LDA, for finger classification tasks). We included in further analysis all trials for which we obtained non-zero predictions from all classifiers.

For the grasp force tasks (grasp force and grasp force + carry), we compared the gated approach to a Wiener cascade for comparison (Glaser *et al* 2020). Briefly, the Wiener cascade performs a linear regression between the neural features (we used 20 factors from Factor Analysis) and the target force. The residuals from that regression are then fit with a nonlinear function (we chose a 3rd order polynomial). For evaluation, we segmented the data into pre-grasp (25th to 75th percentile between start of trial and grasp onset), middle-of-grasp (25th to 75th percentile between grasp onset and grasp offset), and after-grasp (25th to 75th percentile between grasp offset and end of trial) epochs and computed the mean of the predictions within them. We calculated the *R*^2^ between those mean epoch predictions and the corresponding target forces. We also calculated the range of force predictions during the middle-of-grasp epoch, normalized by the mean, as a measure of prediction stability.

For the finger click tasks, we compared the gated approach with standard LDA and calculated the classification accuracy of the held-out predictions across all timepoints of a session. We also found the duration of each click and divided by the cued click duration. This provided a metric to assess how the predictions persisted throughout the intended action duration.

## 3. Results

### 3.1. Motivation for gated decoding approach

**Figure 2.**
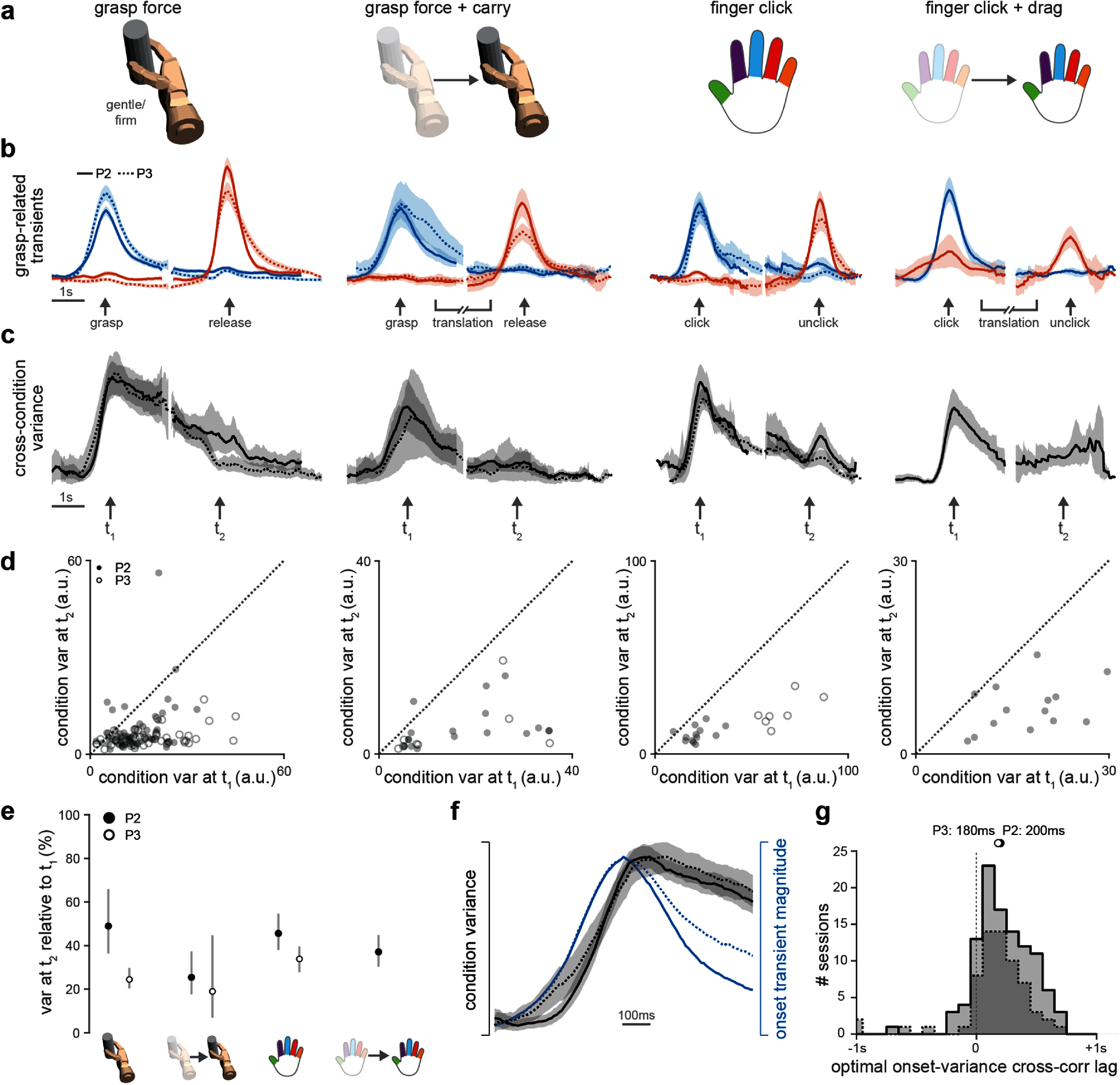
Relationship between nonspecific hand-related transients and specifics of the action. (a) Tasks included grasp force only, grasp force with object carry, finger click, and finger click with drag. (b) Onset (blue) and offset (orange) transients identified for each task, averaged across sessions. (c) Condition-dependent variance in the neural activity as a function of time, aligned to onset and offset components in (b). (d) Scatterplot of condition-dependent neural variance between two timepoints, indicated in (c). (e) Summary across all sessions of each task showing the percentage of condition-dependent neural variance remaining at time *t*_2_ compared to *t*_1_. (f) Temporal evolution of condition-dependent neural variance, averaged across all tasks, superimposed with the onset transient. (g) Histogram of the optimal lag between the condition variance and onset transient for all sessions. Values indicated above indicate the median values across all tasks for both P2 (solid) and P3 (open). Note: shaded areas in (b), (c), and (f), and bars in (e) represent 95% confidence intervals.

Offline analysis of various hand-related tasks (see section 2: Behavioral tasks) revealed consistent dynamic responses and properties in the neural activity. First, as observed previously for simple cursor control with click (Dekleva *et al* 2021), the population response contained consistent and separable transient modes at the time of hand-related action onset (hand grasp/finger press) and offset (grasp/finger release; figure 2(b)). These transients are very similar both across sessions of the same hand action and for different actions, with consistent durations of approximately 500 ms (full width at half maximum; see supplementary figures 1(a)–(d)). We then investigated the amount of information related to grasp force and finger identity relative to the timing of those two transient features. For each session, we aligned the activity on each trial to the average time of onset and offset transient occurrence calculated the variability in neural activity associated with the relevant hand-related feature (force or finger identity; figures 2(b) and (c)). For all four tasks and both participants, we observed a peak in the condition-dependent variability immediately after the peak of the onset transient. This variability then decreased for the remainder of the trial. We summarized this degradation in information within two 100 ms time bins (*t*_1_ centered 200 ms after the peak of the onset transient, *t*_2_ centered 200 ms before the peak of the offset transient). Across all sessions for both participants, we found a significant decrease from *t*_1_ to *t*_2_ in both grasp force-related variability (grasp force only: *p* < 0.001, paired *t*-test; grasp force + carry: *p* < 0.001, paired *t*-test) and finger-related variability (finger click only: *p* < 0.001, paired *t*-test; finger click + carry: *p* < 0.001, paired *t*-test). Overall, we found that the condition-specific variability in neural activity at *t*_2_ was only about 20%–50% of what it was at *t*_1_ (figure 2(e)).

We then analyzed the temporal progression of condition-dependent variance relative to the onset transient (figure 2(f)). Based on the similarity of condition-dependent variance across task types, we combined the onset-aligned responses for all tasks to compute the average traces. On individual sessions, we found the time lag that maximized the correlation between the onset transient and the condition variance traces. The average lag was 220 ms for P2 and 170 ms for P3. The consistency of this relationship across sessions (figure 2(g)) suggests that the onset transient can be used as a reliable detector of more specific hand-related neural responses.

### 3.2. Gated decoder architecture

**Figure 3.**
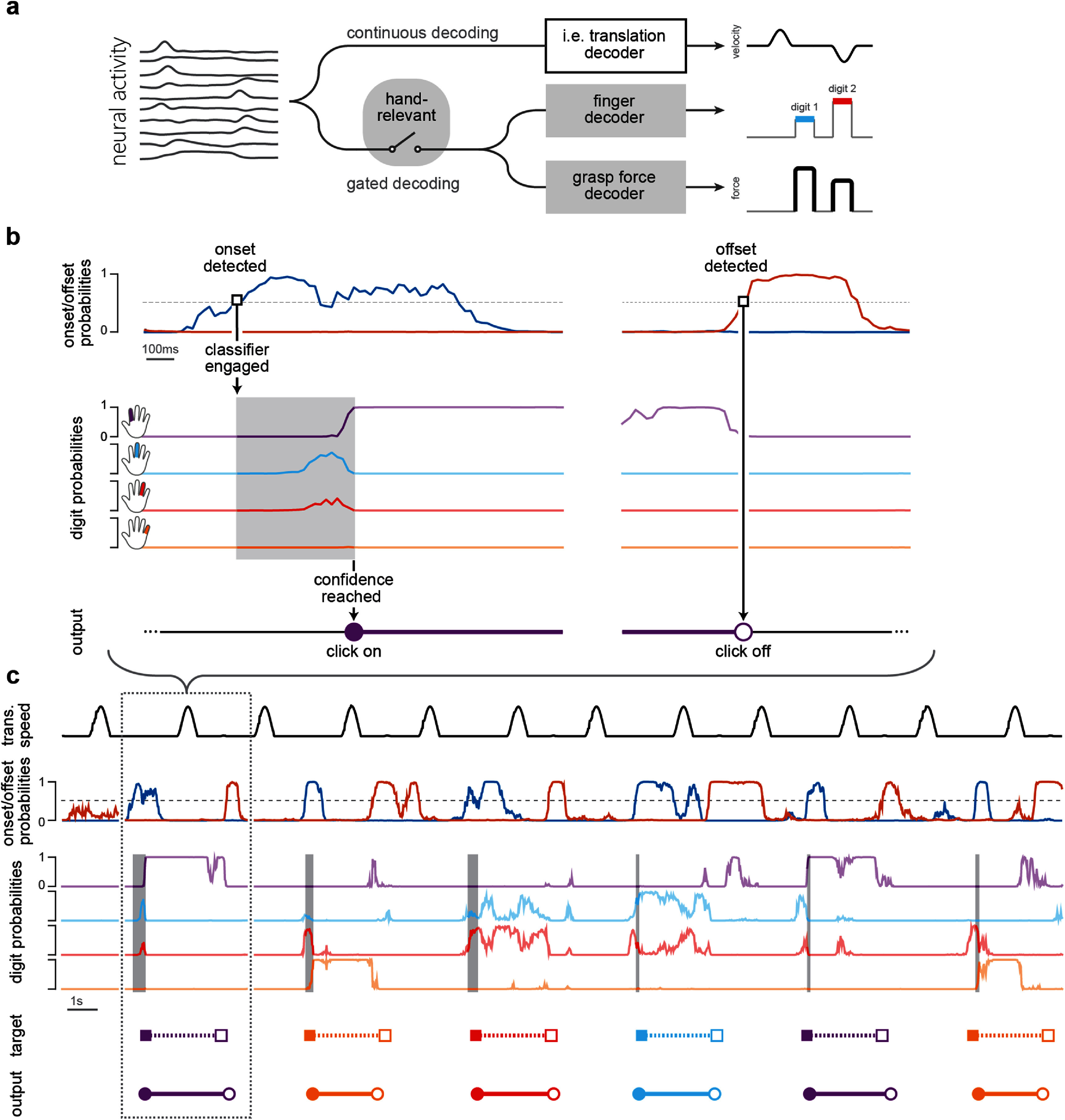
Decoder implementation. (a) Decoder architecture. For the gated decoding approach, specific hand-related decoders are activated only upon detection of general ‘hand-related’ signal. (b) General steps of decoding on a single example trial. (c) Six consecutive trials of brain control performance, including the example trial from (a). Black trace at top represents translation speed during two-dimensional cursor movement. Shaded regions spanning the digit probabilities indicate the time periods during which finger classification took place.

The results from figure 2 indicate that: (1) motor cortex contains transient onset and offset responses for a variety of hand-related actions, (2) the neural variability associated with specific details of that action peaks approximately 150–250 ms after the onset transient (3) the condition-dependent variability decreases over time. From these observations, we developed a gated decoding approach, as outlined in figure 3. The first step of decoding is to identify the occurrence of an onset transient response. Detection of this onset transient then triggers the engagement of a decoder that determines the specifics of the attempted action. The details of this decoder can vary, but for this example we show an approach to finger classification. In our implementation, we applied an LDA classifier at each time point until we reached a pre-specified probability threshold. At this point, the decoder output the resulting estimate and maintained that output until it detected an offset transient response.

### 3.3. Examples of online decoder implementation

**Figure 4.**
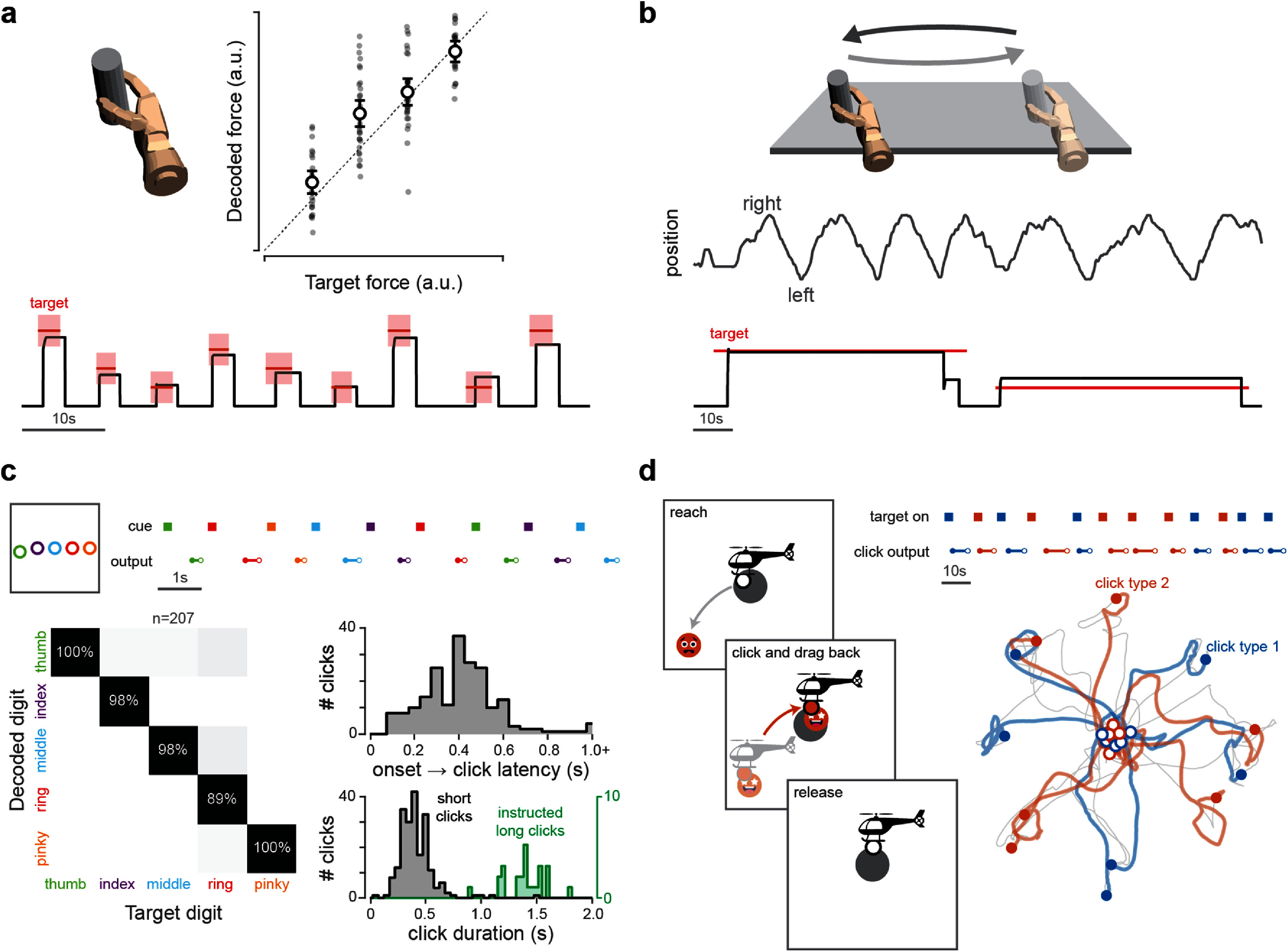
Demonstrations of online decoder use for multiple task types. (a) Grasp force only task. The participant (P3) attempted to achieve four different force targets while receiving line trace feedback of the output force (b) Grasp force plus transport task. Participant (P2) attempted to grasp a virtual object in MuJoCo with one of two force levels and then transport it back and forth across the virtual tabletop for five lengths without dropping. (c) Individual finger decoding. Participant (P3) received a visual cue for one digit at a time and attempted to press and release as quickly as possible. For a subset of trials, we asked the participant to intentionally delay release to demonstrate the flexible control of click duration (bottom histogram, green). (d) Click and drag task with two click types. Participant (P2) attempted to control a cursor from a central target to an outer target (smiley), click on the target and drag it back to center, and then release. Depending on the color of the outer target, the participant needed to perform the click using either an index finger (click type 1: blue) or pinky finger (click type 2: orange).

We have used this gated decoding approach to provide successful online control of grasp force and individual finger actuation across a variety of task contexts and behaviors. Figure 4 highlights a selection of online decoding examples. Figure 4(a) shows the results from a single session of online grasp force control and some example trials. For this grasp force decoding, we used a regression-based implementation of the decoder, which worked slightly differently than the classification-based approach outlined in figure 3. We trained a neural activity-to-force regression model during the periods of high information (i.e. approximately the first second following onset; see figure 2) during calibration trials. During online use, we engaged this regression-based decoder only for the duration of the onset transient. As the transient onset response ended (total duration of transient response after smoothing was approximately one second), we clamped the output at its current value. The output then remained static until detection of the offset transient, at which point we set the force output to zero and opened the hand. The benefit of this clamping behavior is most apparent for tasks involving extended periods of grasp, like the one shown in figure 4(b). For this task, the participant was asked to grasp a virtual cylinder with one of two force levels and repeatedly transport it back and forth across the table (via a separate decoder for arm translation) five times before releasing it. For both force levels, the participant was able to maintain a static force output for over 30 s of concurrent arm translation control.

Figures 4(c) and (d) show online decoding results for example sessions involving finger classification. The task in figure 4(c) was a simple button pressing task, in which the participant received a visual cue to press an on-screen button corresponding to one of the fingers. Upon completion of the press and release, they received another cue (see supplementary video 1 for example). The participant achieved 97% overall finger accuracy, only showing some difficulty with ring finger control. The time from onset transient detection to decoder output was approximately 400 ms (figure 4(c), top histogram), and the average click duration was also approximately 400 ms (figure 4(c), bottom histogram, gray). However, when instructed to maintain the clicks for longer, he was able to easily do so (figure 4(c), bottom histogram, green), showing the flexibility in click duration possible with the onset/offset-based decoding architecture.

Figure 4(d) shows an example session of click-and-drag control with multiple clicks, analogous to left/right buttons on a computer mouse. The task was an extension of the one described in Dekleva *et al* (2021), in which the participant moved a cursor (using BCI control) from the center of the screen to an outer target, then click and drag it back to the center before releasing. Depending on the color of the target (smiley), they needed to use one of two click types, corresponding to either an index or pinky finger press. The highlighted traces in figure 4(c) show the return paths from click on (closed circle) to click off (open circle).

### 3.4. Offline decoder evaluation

**Figure 5.**
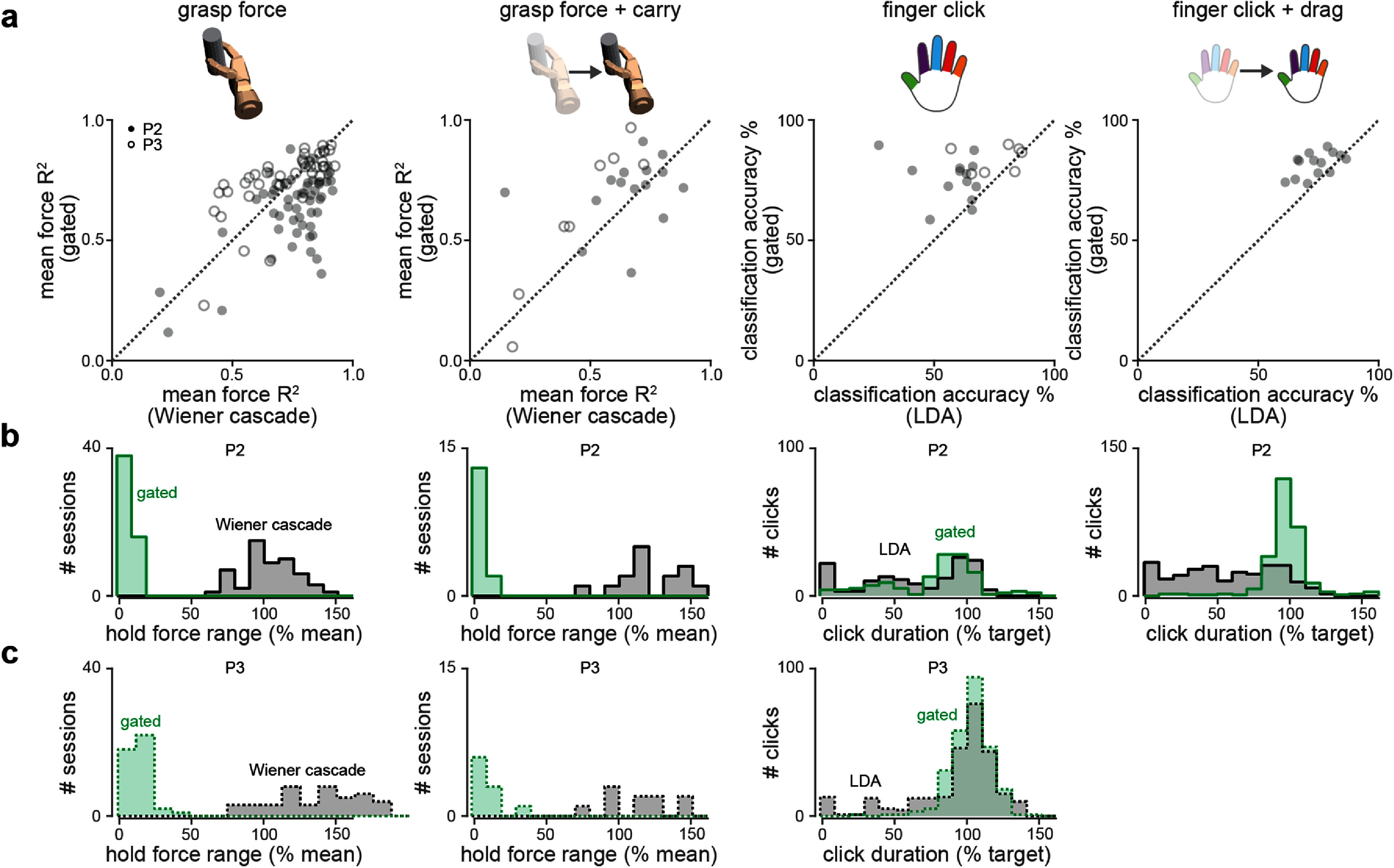
Offline decoder performance comparison. (a) Offline performance metrics using leave-one-out cross validation for all four task types. For grasp force tasks, we computed the *R*^2^ of predictions from the gated approach and a standard Wiener cascade. For finger click tasks, we calculated the overall classification accuracy of predictions from the gated approach and a standard LDA classifier. (b) Additional metrics highlighting the differences in performance between the gated and standard approaches for participant P2. For grasp force, we calculated the average of the total range of forces output during the hold phase (normalized to mean hold force) for both approaches. For finger click, we calculated the duration of all clicks, normalized to the target, or cued, duration. (c) Same as (b) for P3.

The examples in figure 4 show that the gated decoding approach can provide both force and individual digit control for real-world, online BCI control. Additionally, we evaluated cross-validated offline decoding performance on the open-loop task data and compared it to existing, established approaches (figure 5). We found that the average forces decoded throughout a grasp were comparable for the gated approach and a standard Wiener filter (figure 5(a), left). The *R*^2^ values were lower using the gated approach for P2 during the grasp only task (*p* < 0.001, Wilcoxon signed rank test), but higher for P3 (not significantly so, *p* = 0.078, Wilcoxon signed rank test). For the grasp + carry task, *R*^2^ was not significantly different for either participant (P2: *p* = 0.359, P3: *p* = 0.625, Wilcoxon signed rank test). For the finger click tasks, we compared performance of the gated approach to a standard LDA classifier. We found that classification accuracy was higher for the gated approach for P2 in both tasks (finger click: *p*< 0.001, finger click + drag: *p* = 0.001, Wilcoxon signed rank test). For P3, finger click accuracy was higher with the gated approach, but not significantly so (*p* = 0.078, Wilcoxon signed rank test).

Although gated decoding compared favorably to standard approaches on gross performance metrics, these do not fully capture the qualitative differences in output. To provide greater insight into the benefits of a gated decoding approach, we also computed metrics related to stability and output maintenance. For grasp force, we calculated for each grasp the range of forces decoded during the middle half of the grasp period, normalized by the mean force. This provides insight into the variability in decoder output during what should be a static period. We found that for both grasp force tasks, the output of a Wiener cascade during the grasp phase would vary across a range that was approximately equal to its mean (figures 5(b) and (c), left two subplots). However, the gated approach maintained its output during this same period with practically no deviations. Thus, while the mean decoded forces were comparable between the two approaches (figure 5(a)), the gated approach produced a static output while the continuous approach (Wiener filter) varied significantly about the mean (figures 5(b) and (c)). For the finger click tasks, we computed the duration of the click compared to the target value. During the finger click only task, the gated approach significantly outperformed standard LDA in achieving the target click duration for P2 (median 83.2% vs. 65.3%, *p* = 0.002, paired *t*-test) and P3 (median 98.3% vs. 97.3%, *p* < 0.001, paired *t*-test). For the finger click + drag task this improvement was more stark, with the gated approach achieving a median of 91.8% of the target duration compared to 50.1% for standard LDA (*p* < 0.001, paired *t*-test).

## 4. Discussion

We propose here an approach to decoding that contains an end effector-gated architecture. First we identify intention to perform an action using effector-specific (e.g. hand-related) transient neural responses that reliably indicate the initiation and termination of several types of related actions. Subsequently, upon detection of this broad ‘intention to act’ related to the effector in question, we decode the specifics of the action. We then sustain this decoded output until detection of a separate offset-related transient. When tested both offline and online in the control of both grasp force and individual finger presses, it provided high quality control that persisted even with concurrent control of a separate modality (i.e. arm translation).

The specific decoding models for grasp force regression and finger classification used here were quite simple (linear regression for force decoding and LDA for finger classification). Similarly, we used LDA classifiers for onset/offset detection. However, any of these components may be substituted with more complex or nonlinear methods if necessary. The core innovation of the approach is the use of action onset/offset neural transients to trigger start and termination of decoder output, combined with temporally-limited feature decoding at onset. The presence of these transients was consistent across participants and sessions and distributed as latent responses across multiple channels (supplementary materials). The exact details of implementation are flexible, but using effector-specific transients to supplement decoding appears to be a widely applicable approach.

We chose to limit specific feature decoding (i.e. grasp force or finger identity) to a small temporal window after onset detection because we found this period to contain the most informative neural activity regarding precise action parameters (e.g. force or digit identity). Condition-specific variance peaked within 200 ms after the onset transient, which suggests that the onset transient represents a very early motor signature. Even during rapid button presses (supplementary video 1), participants report no noticeable lag between their intent and the decoder output. The onset and offset transients may be similar to (or part of) what has previously been termed the ‘condition-invariant signal’ (CIS; Kaufman *et al* 2016), which was also found to precede condition-specific modulation by a similar amount. For all tasks, the amount of condition-specific variance—essentially the discriminability of different action features—decreased throughout the course of attempted action. There could be multiple reasons for this drop-off in information, though the most likely explanation may be that condition-specific modulation exists across multiple dimensions with different temporal dynamics. For example, both an early transient response and prolonged, sustained response might modulate with grasp force. At grasp initiation, both components would contribute to the overall population-wide grasp force modulation, while later it would be driven entirely by the sustained response. This interpretation is supported by previous experiments using isometric wrist force, which showed that the population response contained a multidimensional set of temporally-distinct force-dependent responses (Dekleva *et al* 2024). In addition to the inherent dynamics of grasp-related activity, much of the drop in condition-specific variance during grasp-and-carry and click-and-drag tasks is likely explained by cross-effector interference. Previous work showed that the addition of arm translation intent during a maintained grasp significantly hampers grasp force decoding (Downey *et al* 2018). Regardless of the exact cause of information loss over time, restricting decoding to a short window around action initiation allowed us to provide stable outputs throughout the entirety of an attempted action (figures 4(b) and (d)).

While our approach of decoding only at action initiation avoided problems due to information drop-off over time, it also meant that on-line adjustments or modifications to the control were essentially impossible. However, a similar transient-based gating approach could be used in combination with continuous within-action decoding. That is, the detection of the onset- and offset-related transients could trigger the engagement and disengagement of a continuous feature decoder. We decided on a more conservative decoder with only a short period of feature decoding because for many real-world use cases of BCI technology (computer cursor control or prosthetic limb control), such instantaneous control is unnecessary and even problematic. For example, while the clamped force output during extended hand grasp (figure 4(b)) prevents fine adjustment, it also prevents unintended object drop. We believe this approach to be most suitable in BCI scenarios that aim to return very basic hand-related functions: computer cursor click, robotic hand grasp, etc. More sensitive decoding approaches are almost certainly needed for future applications requiring fine control and dexterity, but such continuous decoding can still use a transient-based gating approach to prevent unwanted output in the absence of action intent (e.g., as illustrated in figure 1).

The two-level structure of this decoding approach is similar in concept to previous approaches that explicitly switch decoder properties to address nonlinearities arising from neural activity during ‘idle’ and ‘movement’ states (Wu *et al* 2004, Flamary and Rakotomamonjy 2012, Suway *et al* 2013, Wang *et al* 2013, Williams *et al* 2013, Velliste *et al* 2014, Sachs *et al* 2015, Schaeffer and Aksenova 2016a, 2016b). Such methods, for example Switching Linear Decoders (Schaeffer and Aksenova 2016a) also limit feature decoding to relevant time periods, but rely on the segregation of neural data into two states corresponding to action and inaction. Such approaches do allow for better ‘null’ predictions during times of no movement intent but will still suffer from temporal instability when the quality of feature representation changes over the course of an action (see figure 2(c)). Also, it is unclear whether ‘idle’ and ‘action’ states can be reliably differentiated for different effectors simultaneously. The approach presented in this manuscript relies on nuanced transient responses that appear only at the onset and offset of hand actions. This creates highly restricted windows for feature decoding, limiting the influence of temporal variability or effector interactions on decoder output.

The piecewise nature of this decoding approach is also reminiscent of recent neural network-based decoding approaches that employ attentional components (Glaser *et al* 2020, Premchand *et al* 2020, Liu *et al* 2022, Ma *et al* 2022, Okorokova *et al* 2024). Our approach uses the identification of known transient responses to gate the decoder output, in a very tailored and labeled fashion. Neural network approaches may be able to achieve high performance decoding by finding similar solutions and might be able to surpass the performance here by uncovering more complex components in the neural responses. However, neural networks require a large amount of highly varied data to achieve stable, generalized control (Pei *et al* 2022). As a custom-tailored approach, our method requires very little training data (on the order of minutes) and is inherently robust to changes in task context (due to the hand-specific nature of the onset/offset transient responses; figure 3(c)). Thus, we believe this transient-based gated decoding approach provides the robust, reliable control necessary for real-world use of BCI technology.

## 5. Conclusions

BCIs often struggle to generalize across behavioral contexts. The relationship between neural activity and a behavioral variable of interest is highly dynamic and context-dependent, which makes application of static decoding models unreliable for complex, real-world scenarios. Here, we show that population recordings from the motor cortex of human participants contain effector-specific transient responses at the onset and offset of movement intention. These transient responses can be used in a gated decoding architecture, where the output of a specific feature decoder (e.g. grasp force, finger identity) is updated only during a brief window during action initiation and then maintained until action termination. This transient-based approach relinquishes nuanced continuous adjustment in favor of robustness across time and multi-effector behaviors. During online use of a BCI, participants were able to maintain decoded hand-related features over long periods and throughout concurrent arm translation, with a level of stability not possible with standard continuous approaches.

## Data Availability

Deidentified data from this work are posted on DABI, a repository for data related to the National Institutes of Health Brain Research Through Advancing Neurotechnologies Initiative. The data that support the findings of this study are openly available at the following URL/DOI: https://doi.org/10.18120/skf3-y163.

## Code availability

The code to reproduce the main results of this manuscript is publicly available at https://github.com/pitt-rnel/GatedDecoding.

## References

[jneadaa1fbib1] Altan E, Ma X, Miller L E, Perreault E J, Solla S A (2023). Low-dimensional neural manifolds for the control of constrained and unconstrained movements. bioRxiv Preprint.

[jneadaa1fbib2] Azabou M, Arora V, Ganesh V, Mao X, Nachimuthu S, Mendelson M, Richards B, Perich M, Lajoie G, Dyer E (2023). A unified, scalable framework for neural population decoding. Advance Neural Information Processing System.

[jneadaa1fbib3] Dekleva B M, Chowdhury R H, Batista A P, Chase S M, Yu B M, Boninger M L, Collinger J L (2024). Motor cortex retains and reorients neural dynamics during motor imagery. Nat. Hum. Behav..

[jneadaa1fbib4] Dekleva B M, Weiss J M, Boninger M L, Collinger J L (2021). Generalizable cursor click decoding using grasp-related neural transients. J. Neural Eng..

[jneadaa1fbib5] Downey J E, Weiss J M, Flesher S N, Thumser Z C, Marasco P D, Boninger M L, Gaunt R A, Collinger J L (2018). implicit grasp force representation in human motor cortical recordings. Front. Neurosci..

[jneadaa1fbib6] Flamary R, Rakotomamonjy A (2012). Decoding finger movements from ECoG signals using switching linear models. Front. Neurosci..

[jneadaa1fbib7] Flesher S N, Collinger J L, Foldes S T, Weiss J M, Downey J E, Tyler-Kabara E C, Bensmaia S J, Schwartz A B, Boninger M L, Gaunt R A (2016). Intracortical microstimulation of human somatosensory cortex. Sci. Trans. Med..

[jneadaa1fbib8] Gallego J A, Perich M G, Chowdhury R H, Solla S A, Miller L E (2018). A stable, long-term cortical signature underlying consistent behavior. bioRxiv Preprint.

[jneadaa1fbib9] Gao Y, Black M J, Bienenstock E, Wu W, Donoghue J P (2003). A quantitative comparison of linear and non-linear models of motor cortical activity for the encoding and decoding of arm motions.

[jneadaa1fbib10] Georgopoulos A P, Massey J T (1985). Static versus dynamic effects in motor cortex and area 5: comparison during movement time. Behav. Brain Res..

[jneadaa1fbib11] Glaser J I, Benjamin A S, Chowdhury R H, Perich M G, Miller L E, Kording K P (2020). Machine learning for neural decoding. eNeuro.

[jneadaa1fbib12] Kaufman M T, Seely J S, Sussillo D, Ryu S I, Shenoy K V, Churchland M M (2016). The largest response component in the motor cortex reflects movement timing but not movement type. eNeuro.

[jneadaa1fbib13] Kobak D, Brendel W, Constantinidis C, Feierstein C E, Kepecs A, Mainen Z F, Qi X-L, Romo R, Uchida N, Machens C K (2016). Demixed principal component analysis of neural population data. eLife.

[jneadaa1fbib14] Liu F, Meamardoost S, Gunawan R, Komiyama T, Mewes C, Zhang Y, Hwang E, Wang L (2022). Deep learning for neural decoding in motor cortex. J. Neural Eng..

[jneadaa1fbib15] Ma X, Qiu S, He H (2022). Time-distributed attention network for EEG-based motor imagery decoding from the same limb. IEEE Trans. Neural Syst. Rehabil. Eng..

[jneadaa1fbib16] Naufel S, Glaser J I, Kording K P, Perreault E J, Miller L E (2019). A muscle-activity-dependent gain between motor cortex and EMG. J. Neurophysiol..

[jneadaa1fbib17] Okorokova E V, Sobinov A R, Downey J E, He Q, van Driesche A, Satzer D, Warnke P C, Hatsopoulos N G, Bensmaia S J, Guger C, Allison B, Rutkowski T M, Korostenskaja M (2024). May the force be with you: biomimetic grasp force decoding for brain controlled bionic hands. brain-Computer Interface Research: A State-of-the-Art Summary 11 edn.

[jneadaa1fbib18] Pei F (2022). Neural latents benchmark’21: evaluating latent variable models of neural population activity.

[jneadaa1fbib19] Premchand B, Toe K K, Wang C, Shaikh S, Libedinsky C, Ang K K, So R Q (2020). Decoding movement direction from cortical microelectrode recordings using an LSTM-based neural network.

[jneadaa1fbib20] Qi Y, Liu B, Wang Y, Pan G (2019). Dynamic ensemble modeling approach to nonstationary neural decoding in brain- computer interfaces. https://proceedings.neurips.cc/paper/2019/hash/3f7bcd0b3ea822683bba8fc530f151bd-Abstract.html.

[jneadaa1fbib21] Sachs N A, Ruiz-Torres R, Perreault E J, Miller L E (2015). Brain-state classification and a dual-state decoder dramatically improve the control of cursor movement through a brain-machine interface. J. Neural Eng..

[jneadaa1fbib22] Schaeffer M-C, Aksenova T (2016b). Switching Markov decoders for asynchronous trajectory reconstruction from ECoG signals in monkeys for BCI applications. J. Physiol..

[jneadaa1fbib23] Schaeffer M-C, Aksenova T, Villa A E P, Masulli P, Rivero A J (2016a). Hybrid trajectory decoding from ECoG signals for asynchronous BCIs. Artificial Neural Networks and Machine Learning—ICANN 2016.

[jneadaa1fbib24] Shalit U, Zinger N, Joshua M, Prut Y (2012). Descending systems translate transient cortical commands into a sustained muscle activation signal. Cereb. Cortex.

[jneadaa1fbib25] Suway S B, Tien R N, Jeffries S M, Zohny Z, Clanton S T, McMorland A J C, Velliste M (2013). Resting state detection for gating movement of a neural prosthesis.

[jneadaa1fbib26] Velliste M, Kennedy S D, Schwartz A B, Whitford A S, Sohn J-W, McMorland A J C (2014). Motor cortical correlates of arm resting in the context of a reaching task and implications for prosthetic control. J. Neurosci..

[jneadaa1fbib27] Wang P T (2013). State and trajectory decoding of upper extremity movements from electrocorticogram.

[jneadaa1fbib28] Williams J J, Rouse A G, Thongpang S, Williams J C, Moran D W (2013). Differentiating closed-loop cortical intention from rest: building an asynchronous electrocorticographic BCI. J. Neural Eng..

[jneadaa1fbib29] Wu W, Black M J, Mumford D, Gao Y, Bienenstock E, Donoghue J P (2004). Modeling and decoding motor cortical activity using a switching Kalman filter. IEEE Trans. Biomed. Eng..

[jneadaa1fbib30] Xiao J, Liu R, Dyer E L (2023). GAFormer: enhancing timeseries transformers through group-aware embeddings. https://openreview.net/forum?id=c56TWtYp0W.

[jneadaa1fbib31] Ye J, Collinger J, Wehbe L, Gaunt R (2023). Neural Data Transformer 2: multi-context pretraining for neural spiking activity. Advances in Neural Information Processing Systems.

[jneadaa1fbib32] Zou S, Wang S, Zhang J, Zong C (2021). Towards Brain-to-Text Generation: Neural Decoding with Pre-trained Encoder-decoder Models. https://openreview.net/forum?id=13IJlk221xG.

